# The highest clinical trial failure rate for any disease in modern medicine: How both amyloid- and non-amyloid-targeting therapies failed to alter disease course in Alzheimer's disease

**DOI:** 10.17879/freeneuropathology-2026-9650

**Published:** 2026-08-27

**Authors:** Michael A.S. Guth

**Affiliations:** 1 Institute for Neuroplasticity Research, 116 Oklahoma Ave, Oak Ridge, TN 37830-8604, USA

**Keywords:** Alzheimer's disease, Clinical trials, Amyloid hypothesis, Drug development, CADRO, Disease-modifying therapy, Go/No Go decisions

## Abstract

**Background:** Alzheimer's disease drug development has produced a 99.6 percent failure rate for disease-modifying compounds (244 compounds tested 2002–2012, one regulatory approval; Cummings et al., 2014; Cummings, 2018)—the most sustained and costly therapeutic failure in the history of modern medicine. The field's standard explanation is that the biology is intractable. The present analysis offers a different and more instructive diagnosis: the failure was not primarily scientific. It was institutional.

**Objective:** To characterize the structural and institutional forces that sustained investment in repeatedly ineffective mechanisms of action across 567 Phase 2 and Phase 3 Alzheimer's trials from 2010 to 2026, and to determine whether peer-reviewed alternative pathogenic frameworks were available at the time each major Go/No Go decision was made.

**Methods:** Trials were identified from ClinicalTrials.gov using the following criteria: primary diagnosis of Alzheimer's disease or mild cognitive impairment attributed to Alzheimer's disease; Phase 2 or Phase 3; status of terminated or completed; primary completion date between January 1, 2010 and May 4, 2026. After exclusion of 70 trials not classifiable by mechanism of action, a final analytic dataset of 497 trials was classified by primary mechanism using the Common Alzheimer's Disease Research Ontology (CADRO). A temporal alignment analysis was conducted for the 12 largest trials by enrollment (N ≥ 1,300), mapping each trial's initiation date against the publication dates of peer-reviewed alternative mechanistic frameworks available at the time of trial launch.

**Results:** Amyloid-targeting approaches accounted for approximately 31 percent of the 497 classified trials, nearly all of which failed to demonstrate clinical efficacy. Among the 12 largest trials, 11 targeted amyloid pathology and 10 were terminated for futility or lack of efficacy. All 12 trials were initiated after the publication in high-impact journals of peer-reviewed alternative frameworks, including the TREM2 neuroinflammation pathway, the antimicrobial protection hypothesis, the periodontitis-associated bacterial pathogenesis hypothesis, and the GLP-1 metabolic pathways. The post-2022 period revealed that genuinely novel non-amyloid mechanisms—TREM2 agonism, GLP-1 receptor stimulation, filamin A modulation, and progranulin/sortilin restoration—also failed to produce clinical benefit at scale despite confirmed target engagement. Three establishment forces are identified as operative causes: regulatory path dependency, institutional entrenchment within grant and editorial review panels, and shareholder-driven sunk-cost pressures.

**Conclusion:** The 567-trial record constitutes a structural verdict on a model of drug development that was never designed to solve a problem of this complexity. The uniformity of negative results across all mechanism categories—amyloid, tau, neuroinflammation, metabolic, and synaptic—points toward deeper problems of disease definition, intervention timing, and endpoint validity that institutional and financial pressures have prevented the field from confronting. The Dominantly Inherited Alzheimer Network (DIAN) biomarker data demonstrates that pathology precedes symptoms by 15–25 years; the 2025 DIAN Trials Unit (DIAN-TU) extension provides the first evidence that treatment before symptom onset may delay dementia. Prevention trials in asymptomatic at-risk populations represent the only strategy the accumulated trial record has not yet refuted.

## Key points

• **Institutional failure, not hard biology alone:** The 0.4 percent clinical development success rate across Alzheimer's disease-modifying compounds reflects not only the intractability of the biology but three compounding institutional forces— regulatory path dependency, entrenchment within peer review and grant panels, and shareholder-driven sunk-cost pressures—that systematically overrode accumulating negative evidence (Cummings et al., 2014; Cummings, 2018; Kim et al., 2022).

• **The temporal alignment finding:** Every one of the 12 largest trials in this dataset was initiated after peer-reviewed alternative pathogenic frameworks—including the TREM2 neuroinflammation pathway, the antimicrobial protection hypothesis, and GLP-1 metabolic pathways—had already appeared in high-impact journals. None of these frameworks was incorporated into trial design, endpoint selection, or Go/No Go deliberations (Guerreiro et al., 2013; Soscia et al., 2010; Heneka et al., 2015; Hölscher, 2014).

• **The BACE inhibitor evidence:** Peer-reviewed mechanistic concerns about BACE1's physiological necessity for axon guidance and synaptic integrity were published before the third and fourth BACE inhibitor trials were launched; elenbecestat's adverse event profile—worse than placebo— confirmed those predictions, documenting that the mechanistic concerns had been available in the published literature at the time of the Go decisions (Vassar, 2014).

• **The post-2022 challenge:** Genuinely novel non-amyloid mechanisms—TREM2 agonism (AL002/INVOKE-2), GLP-1 receptor stimulation (EVOKE/EVOKE+), filamin A modulation (REFOCUS-ALZ), and progranulin/sortilin restoration (nivisnebart)—also failed at scale with confirmed target engagement. This establishes that the problem is not mechanism selection alone but disease definition, intervention timing, and endpoint validity (Bateman et al., 2012; Lanctôt et al., 2025; Li et al., 2026).

• **The timing imperative:** The DIAN longitudinal biomarker dataset demonstrates that pathological changes detectable by cerebrospinal fluid and PET begin 15–25 years before symptom onset. The 2025 DIAN-TU open-label extension provides the first evidence that anti-amyloid treatment before symptom onset may delay dementia—using the same antibody that failed when given after symptoms appeared. Prevention trials in asymptomatic at-risk populations, with long-term follow-up (> 5 years), represent the only strategy the 567-trial record has not yet refuted (Bateman et al., 2012; Bateman et al., 2025; Guth, 2026).

## Introduction

Alzheimer's disease impacts approximately six million Americans and 35 million people worldwide, with predictions indicating these numbers could triple by 2050 in the absence of effective disease-modifying treatment. The human toll is substantial. The costs to the pharmaceutical industry and public research funding are considerable. Moreover, the return on that investment, measured by drugs that truly halt the disease, remains essentially nonexistent. Two approved drugs—lecanemab and donanemab—modestly slow decline in carefully selected, early-stage patients over a limited period. They do not stop the disease. They do not reverse it. They target biomarker surrogates—protofibrils and pyroglutamate-3 amyloid beta (pGlu3-Aβ), respectively—whose connection to individual-level clinical benefit has not been confirmed.

Since 2000, the clinical development success rate for disease-modifying Alzheimer's compounds in Phase 2 and Phase 3 trials has been estimated at approximately 0.4 percent, based on an analysis of 244 compounds tested between 2002 and 2012 that yielded a single regulatory approval (Cummings et al., 2014; Cummings, 2018). A subsequent analysis covering the period since 2003 found a somewhat higher but still exceptionally low success rate of 2.0 percent—two successes (aducanumab and oligomannate) against 98 failed compounds (Kim et al., 2022). Among amyloid-targeting compounds specifically, the success rate is effectively zero when measured by durable clinical benefit. Lecanemab and donanemab have gained regulatory approval based on amyloid clearance, but the clinical significance of the benefit is dubious. Lanctôt et al. (2025) demonstrated that even complete halting of disease progression would not meet published minimal clinically important difference thresholds for the Clinical Dementia Rating scale—Sum of Boxes (CDR-SB) in mild Alzheimer’s disease (AD) dementia. No published case reports describe patients regaining functional abilities. Statistical significance is not clinical significance. The persistent difficulty in demonstrating clinical benefit via amyloid clearance raises the question of whether Aβ may serve a protective, rather than purely pathogenic, role—a sequestration hypothesis explored elsewhere (Guth, 2026).

The observation that prompted this article is not the failure rate per se but rather what it reveals about the decisions that produced it. The full universe of 567 Phase 2 and Phase 3 Alzheimer's trials from 2010 to 2026 reveals not a story of scientists heroically fighting difficult biology. Instead, the history of these 567 trials illustrates repeated private industry decisions to treat Alzheimer's disease by focusing on removal of Aβ plaques, even when that approach led to failure by competing firms.

The first chapter is familiar: an extraordinary concentration of resources in amyloid-targeting mechanisms—beta-site amyloid precursor protein cleaving enzyme (BACE) inhibitors, anti-amyloid antibodies, active vaccines, gamma-secretase inhibitors—that produced 153 trials, or approximately 31 percent of the entire dataset, with a nearly complete lack of clinical efficacy. The second chapter is less often told: 321 trials across diverse mechanisms of action— neuroinflammation, metabolic, vascular, synaptic, progranulin/sortilin—that also failed, almost without exception.

The full dataset of 567 Phase 2 and Phase 3 Alzheimer's trials from 2010 to 2026 reveals a field that is less monolithic than its critics often suggest—and more uniformly unsuccessful than its defenders typically acknowledge. The first table in the Results section presents the distribution of mechanisms across the dataset. The richness of this mechanistic diversity is noteworthy. The uniformity of the negative results across all categories justifies medical practitioners' and the public's concern.

This article addresses two questions. First, why did the field continue to commit resources at amyloid scale despite an accumulating record of negative results and the availability of published alternative frameworks? Second, why, when the field eventually diversified mechanistically, did the alternatives also fail? These are not the same questions. The first implies institutional decision-making and financial entrenchment. The second implicates something more fundamental: the possibility that the field's entire intervention framework—the patient populations, the timing, the endpoints—is miscalibrated regardless of mechanism. Alternatively, after a century of investigation, the field may still have no better understanding of the root cause(s) for a collection of syndromes now called Alzheimer's disease.

**Figure 1:**
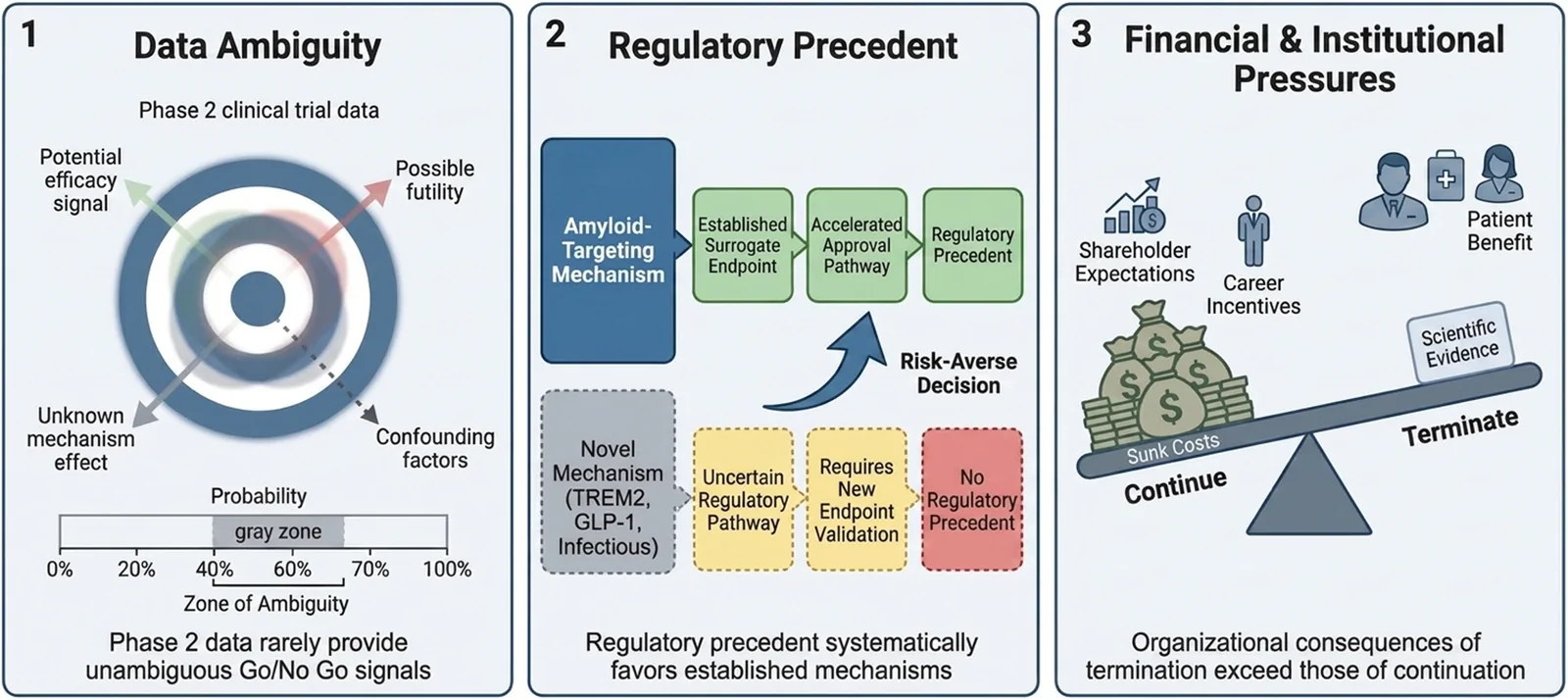
**Figure 1**. **Stylized illustration of data ambiguity, regulatory path dependency, and institutional pressures influencing Alzheimer's disease Go/No Go decision meetings.** Phase 2 data frequently produce ambiguous signals (**Panel 1**), creating interpretive latitude that allows organizational pressures to fill the evidentiary gap. Regulatory precedent for amyloid clearance as a surrogate endpoint created path-dependent incentives favoring established mechanisms over novel approaches (**Panel 2**). Sunk-cost pressures and asymmetric consequences of termination versus continuation systematically biased decisions toward continuation (**Panel 3**). This figure is a schematic representation of structural factors affecting Go/No Go decisions, not an allegation of misconduct.

The 567 clinical trials required 567 Go/No Go decisions and data review meetings. In those meetings, one question above all others should have been asked and answered honestly: does the Phase 2 clinical signal for this mechanism justify committing thousands of patients and tens or hundreds of millions of dollars to a Phase 3 trial, given everything the accumulated trial record has already shown about this mechanism class? The evidence, reconstructed from the public record, suggests that this question was not being asked rigorously—or that the answer was being overridden by financial and organizational pressures unrelated to science. **Figure 1** illustrates the data uncertainty, regulatory path dependency, and the Food and Drug Administration (FDA) surrogate endpoint problem confronted in many Go/No Go decision meetings.

## Background: From Alzheimer's microscope to the amyloid framework

One hears sometimes—from clinicians frustrated with the field's record—that it is unreasonable to build a century of drug development on the 1906 examination of a single patient's brain by Alois Alzheimer. The frustration is understandable. But the characterization misidentifies the origin of the problem.

Alois Alzheimer's 1906 observation was legitimate science for its era. Using Bielschowsky silver staining—a newly developed technique—he identified the pathological features of what would become a named disease: plaques, tangles, and profound neuronal loss in the cortex. He was not drawing sweeping causal conclusions from one case; he was describing, for the first time, a histopathological signature of a condition that had been observed clinically but never characterized. And he was not dogmatic. In 1911, he described cases with extensive plaques but few tangles, acknowledging that the pathological picture was more variable than a single canonical case would suggest (Alzheimer, 1911; Möller & Graeber, 1998).

The founding error of modern Alzheimer's drug development was not the 1906 examination. It was the field's subsequent interpretation of the 1992 Hardy and Higgins paper in Science, which formalized what became known as the amyloid cascade hypothesis (Hardy and Higgins, 1992). The accumulation of amyloid-beta peptides in the brain was proposed as the central, causative event in Alzheimer's pathogenesis, with neurofibrillary tangles, cell loss, vascular damage, and dementia all following as downstream consequences. That was a hypothesis. It was treated almost immediately as a settled framework. Within a few years, the implicit assumption in the field had become that researchers not studying amyloid were not studying Alzheimer's disease. Even the hypothesis originator, John Hardy, would later acknowledge that the field had “this idea of a magic bullet” that did not materialize (Lourenco, 2022). The man whose name the disease bears was notably humble and cautious about what his 1906 findings actually proved; his successors were not.

What makes this history particularly significant is what Hardy and Higgins actually wrote in 1992. They suggested that “other causes of Alzheimer's act by initially triggering amyloid-beta peptide deposition” and that “amyloid deposition occurs as an acute response to neuronal injury in both man and animals” (Hardy and Higgins, 1992). The founders of the hypothesis explicitly allowed for the possibility that amyloid is a response rather than a cause. The field largely ignored that escape hatch for thirty years.

A subsequent study of citation practices for the Hardy and Higgins paper across 445 articles found that 62 percent cited it with neutral attitudes and 35 percent with positive attitudes, with only a small minority expressing skepticism, across the entire period from 1992 to 2019—despite the absence of successful clinical trials confirming the causal hypothesis (Daly et al., 2020). The field did not update its confidence in the hypothesis as the trial failures accumulated. Each negative trial was interpreted as a trial design problem, a patient selection problem, or a timing problem. The hypothesis itself was not systematically reconsidered.

Meanwhile, researchers were finding considerably more in Alzheimer's brains than amyloid and tau. Neuroinflammation and microglial activation were documented early but classified as “secondary” to amyloid—a classification that was an assumption, not a finding (Akiyama et al., 2000). Alpha-synuclein co-pathology was observed but called a “comorbidity” (Bellomo et al., 2024). A 2013 study in Brain found that one-third of non-demented individuals had the same amyloid and tau burden as diagnosed Alzheimer's patients, a direct challenge to the sufficiency of the canonical pathology for producing dementia. The lead author concluded that amyloid-beta plaque deposition and tangle formation do not inevitably result in dementia and neuronal damage in all individuals (Perez-Nievas et al., 2013). The implication is that something else—neuroinflammation, synaptic resilience, vascular health—determines who becomes symptomatic and who does not.

The mechanism of the field's entrenchment was not primarily intellectual. It was financial and institutional. NIH grant review panels were disproportionately populated by researchers whose careers were built on the amyloid framework. Journal editorial boards were similarly constituted. Researchers working on infectious hypotheses, primary neuroinflammation, or metabolic frameworks reported systematic difficulty obtaining funding. This is the sociology of science functioning as it normally does—and producing exactly the outcome one would predict from such a structure when the dominant hypothesis turns out to be insufficient.

## Methods

### Trial dataset

Trials were identified from the ClinicalTrials.gov database using the following inclusion criteria: primary diagnosis of Alzheimer's disease or mild cognitive impairment attributed to Alzheimer's disease; Phase 2 or Phase 3; study status of either TERMINATED or COMPLETED; primary completion date between January 1, 2010 and May 4, 2026. Trials were excluded if they were Phase 1, withdrawn, suspended, or not yet recruiting. This search yielded 567 trials.

Seventy trials were subsequently excluded from the mechanism-stratified analysis on the following grounds: duplicate registry entries for the same trial; trials in which the primary intervention was a dietary supplement rather than a pharmaceutical or biological agent; trials testing exclusively symptomatic or caregiver-support endpoints with no pharmacological intervention; and trials for which mechanism of action could not be determined from any available registry field, drug name, or published protocol. The mechanism-stratified analysis therefore proceeds from a final analytic dataset of 497 trials. The figure of 567 is retained throughout the text where the reference is to the total universe of registered Phase 2 and Phase 3 Alzheimer's trials meeting the inclusion criteria, without regard to classifiability.

For the detailed Go/No Go analysis, we focused on the 12 largest trials by enrollment (N ≥ 1,300). This threshold was chosen to capture trials representing the largest financial investments and therefore the decisions with the greatest consequences. The 12-trial analysis is a case study within the broader dataset, not a substitute for it. The 567-trial dataset provides the epidemiological landscape; the 12-trial analysis provides the temporal and mechanistic argument about what was known at the moment each Go decision was made.

### Mechanism classification

Each of the 497 analyzed trials was classified by primary mechanism of action using the Common Alzheimer's Disease Research Ontology (CADRO) (Cummings et al., 2023). Categories included: amyloid-beta (with sub-categories for BACE inhibitors, anti-amyloid antibodies, active vaccines, and gamma-secretase inhibitors); tau (anti-tau antibodies and aggregation inhibitors); neurotransmitter (cholinergic, glutamatergic, and histaminergic agents); neuroinflammation (triggering receptor expressed on myeloid cells 2 (TREM2)-targeting agents, nonsteroidal anti-inflammatory drugs (NSAIDs), and cytokine modulators); metabolic/mitochondrial (insulin sensitizers, peroxisome proliferator-activated receptor (PPAR) agonists, and glucagon-like peptide-1 (GLP-1) receptor agonists); neuroprotection/synaptic; progranulin/sortilin; vascular/cerebrovascular; and other/novel/combination.

The 'Other' category, encompassing 118 trials in this dataset, is not a homogeneous residual. It reflects genuine mechanistic diversity—from stem cell approaches and antioxidant therapies to lifestyle interventions and combination regimens. The failure of this category alongside the amyloid-centric core of the dataset is one of the most important empirical facts in the analysis.

### Alternative framework analysis

For each of the 12 largest trials, a systematic literature search was conducted to identify whether a mechanistic framework other than the one being tested had been proposed as a primary driver of Alzheimer's pathology prior to the trial's start date. Frameworks were included only if they: (1) proposed a causal pathway distinct from the mechanism being tested; (2) were published in a peer-reviewed journal; and (3) appeared before the trial's first posted date on ClinicalTrials.gov.

This 'alternative framework available at trial initiation' assessment is but one of this article's analytical contributions. It addresses the question: at the moment the Go decision was made, what evidence was available in the peer-reviewed literature that might have informed a different decision? Frameworks meeting the inclusion criteria included the antimicrobial protection hypothesis (Soscia et al., 2010; Kumar et al., 2016), the TREM2 neuroinflammation pathway (Guerreiro et al., 2013), the periodontitis-associated bacterial pathogenesis hypothesis (Singhrao et al., 2014), the BACE inhibitor physiological risk prediction (Vassar, 2014), the consolidated neuroinflammation hypothesis (Heneka et al., 2015), and the GLP-1 metabolic and neuroinflammatory pathway (Hölscher, 2014).

## Results

### The 567-trial landscape: A field that has tested widely and advanced little

**Figure 2:**
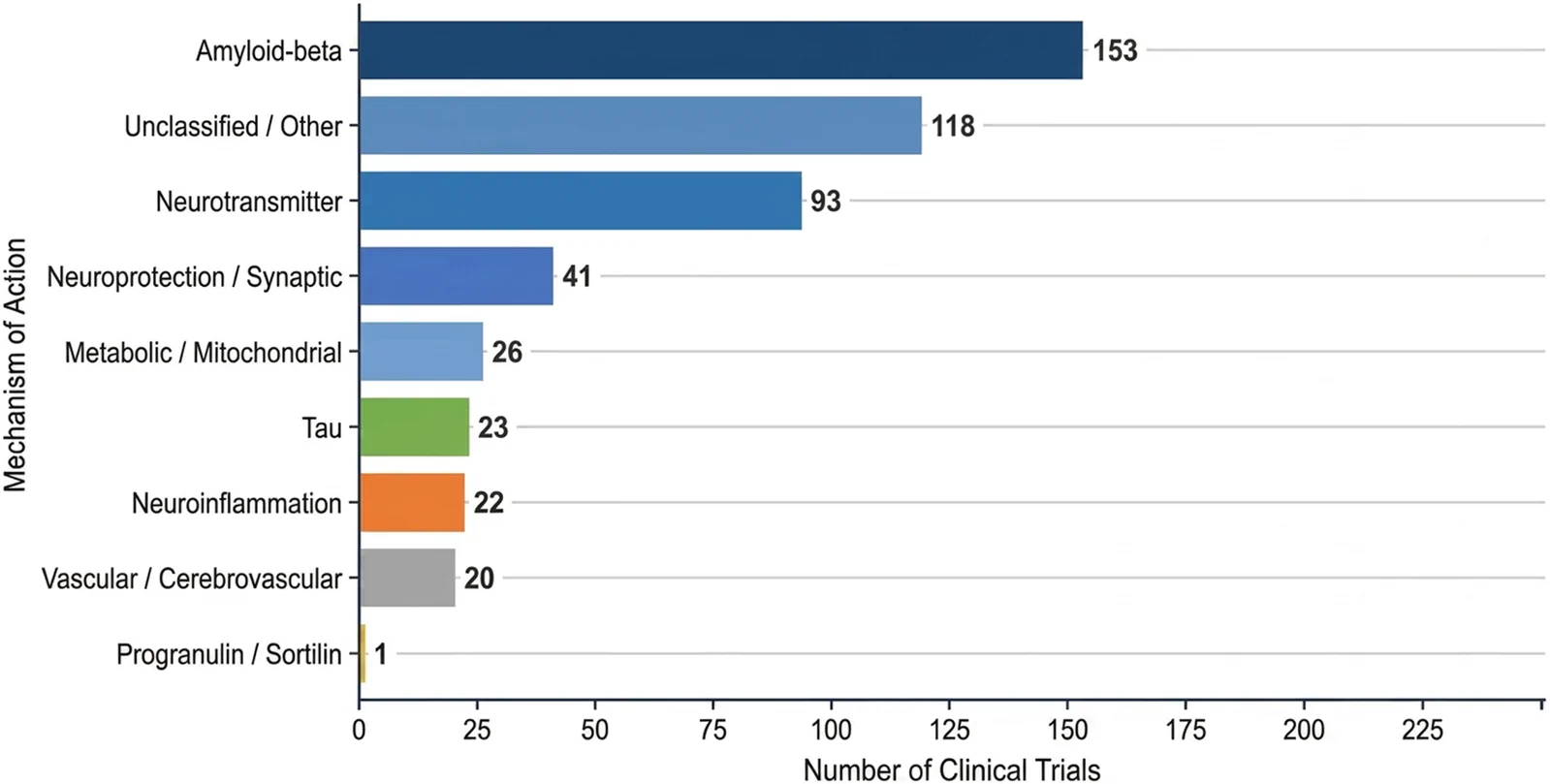
**Figure 2**: **Distribution of Alzheimer's disease clinical trials by mechanism of action (N = 497, 2010–2026).** Horizontal bar chart showing the number of Phase 2 and Phase 3 Alzheimer's disease trials by primary mechanism of action, classified using the Common Alzheimer's Disease Research Ontology (CADRO) taxonomy. Amyloid-targeting mechanisms (153 trials, 30.8 percent) represent the largest single category and exceed all other individual mechanism classes. The "Other / Novel / Unclassified" category (118 trials, 23.7 percent) reflects genuine mechanistic diversity, including stem cell approaches, antioxidant therapies, lifestyle interventions, and combination regimens. The remaining categories—neurotransmitter (93 trials), neuroprotection/synaptic (41), metabolic/mitochondrial (26), tau (23), neuroinflammation (22), vascular/cerebrovascular (20), and progranulin/sortilin (1)—demonstrate that the field tested broadly across mechanism classes. The uniformity of negative results across all categories, despite this mechanistic diversity, indicates that the problem is not mechanism selection alone. Termination rates across categories range from 10 percent to 30 percent (excluding Progranulin/Sortilin with an N=1). Source: ClinicalTrials.gov (497 Alzheimer's interventional trials).

The full dataset of 567 Phase 2 and Phase 3 Alzheimer's trials from 2010 to 2026 reveals a field that is less mechanistically monolithic than its critics often suggest—and more uniformly unsuccessful than its defenders acknowledge. **Table 1** presents the mechanism distribution, and **Figure 2** displays the same distribution visually as a bar chart.

**Table 1:** 

**Table 1:** Phase 2/3 Alzheimer's disease trials by mechanism of action (N = 497, 2010–2026)

**Mechanism of Action**	**Trials (N)**	**Share**	Termination Rate*	**Representative Agents / Notes**
Amyloid-beta (BACE inhibitors, anti-Aβ mAbs, active vaccines, γ-secretase inhibitors)	153	30.8 %	30 %	Solanezumab, verubecestat, lanabecestat, elenbecestat, aducanumab, gantenerumab, crenezumab, bapineuzumab, CAD106, BMS-708163, LY3202626, ALZ-801
Tau (anti-tau mAbs, tau vaccines, aggregation inhibitors, GSK3β)	23	4.6 %	26 %	Gosuranemab, tilavonemab, semorinemab, posdinemab, LMTM/TRx0237, tideglusib, bepranemab, AADvac1, ACI-35
Neurotransmitter (cholinergic, glutamatergic, histaminergic, dopaminergic, PDE inhibitors)	93	18.7 %	23 %	Symptomatic management only; no disease modification demonstrated. Donepezil, memantine, idalopirdine, intepirdine (RVT-101), dimebon, ABT-126, EVP-6124, BI 425809, SAGE-718
Neuroinflammation (NSAIDs, cytokine modulators, TREM2 agonists, RAGE inhibitors, TNFα)	22	4.4 %	27 %	AL002 (TREM2/INVOKE-2), azeliragon (RAGE), XPro1595 (TNFα), CHF 5074, masitinib, GV-971 (sodium oligomannate), ALZT-OP1
Metabolic / Mitochondrial (insulin sensitizers, PPAR agonists, GLP-1 agonists, mTOR)	26	5.2 %	15 %	Pioglitazone, intranasal insulin, semaglutide (EVOKE/EVOKE+), NE3107, T3D-959, rapamycin/sirolimus, AC-1204 (ketogenic MCT)
Neuroprotection / Synaptic (BDNF/NGF, filamin A, sigma-2, stem cells, PKC modulators)	41	8.2 %	17 %	Simufilam (REFOCUS-ALZ), CT1812, bryostatin, ANAVEX2-73, ATH-1017, cerebrolysin, LM11A-31, IGIV/albumin, mesenchymal stem cells
Progranulin / Sortilin Pathway (lysosomal pathway, microglial maintenance)	1	0.2 %	0 %	AL001 (latozinemab). Nivisnebart (GSK/ Alector) terminated April 2026—not yet in dataset. Undercounted; category emerged ~2021.
Vascular / Cerebrovascular (antihypertensives, statins, omega-3, NO pathway)	20	4.0 %	10 %	Nilvadipine, losartan, telmisartan, sildenafil, simvastatin, perindopril, L-arginine, omega-3/DHA/EPA, tetrahydrobiopterin
Other / Novel / Unclassified (proprietary compound codes, combination regimens, novel mechanisms)	118	23.7 %	16 %	Includes proprietary codes unresolvable by string matching (BI 409306, ABBV-552, ORM-12741, TB006). Stem cell, antioxidant, lifestyle, and combination regimens. Category is heterogeneous.

Progranulin count (N = 1) reflects dataset lag—category expanded significantly 2022–2026. “Other” includes trials whose mechanism cannot be identified from drug name alone (proprietary compound codes).

Three features of this distribution demand emphasis. First, amyloid-targeting mechanisms in aggregate account for approximately 31 percent of the entire dataset. Amyloid far exceeds any other single target class and represents a concentration of resources without parallel in any other disease area with a comparable failure record. Second, the approximately 321 trials outside the amyloid and tau categories represent genuine mechanistic diversity. This is not a story of scientists who refused to think beyond amyloid; it is a story of a field that thought broadly and still failed comprehensively. Third, and this is the finding that should arrest the reader, the termination rates across virtually every mechanism category are substantial, and the clinical efficacy rate—the proportion of drugs that ultimately demonstrate disease-modifying benefit—approaches zero across all categories.

The implications of that third point are severe. If the problem were simply amyloid-centric, one would expect the non-amyloid trials to perform differently. They do not. The field has tested amyloid clearance, tau reduction, neuroinflammation suppression, GLP-1 receptor stimulation, TREM2 microglial activation, filamin A modulation, progranulin restoration, vascular protection, and synaptic enhancement—and found that none of them, as currently operationalized in Phase 3 trials, produces durable clinical benefit. The question that the 567-trial dataset demands is not 'which mechanism should we try next?' It is 'are we intervening at the right time, in the right patients, and measuring the right outcomes?' If the piecemeal approach of private industry has failed to crack the root causes of Alzheimer's disease after three decades of amyloid-targeted development, the failure is itself a diagnosis: the current incentive structure is not capable of producing the answer. The complexity of the problem and the depth of the institutional failure documented here suggest that what is required is not a better drug candidate— it is a fundamentally different model of inquiry.

### The 12 largest trials: What the Go/No Go decisions actually rested on

Within the 567-trial dataset, the 12 largest trials by enrollment (N ≥ 1,300) represent a disproportionate share of the total financial investment. **Table 2** presents their characteristics alongside the peer-reviewed alternative frameworks that were already published at the time each trial was initiated.

Of the 12 trials, 11 (92 percent) targeted amyloid pathology. This included five BACE inhibitor trials across three drugs (two lanabecestat trials, one elenbecestat trial, two verubecestat trials) and six anti-amyloid monoclonal antibody trials across three drugs (two solanezumab trials, three aducanumab trials, one gantenerumab trial). The sole exception was pioglitazone—the largest single trial in the dataset at 3,494 participants—which also failed to meet its primary endpoint. Pioglitazone is an oral antidiabetic belonging to the thiazolidinedione (or "glitazone") class, commonly known as insulin sensitizers.

Ten of 12 trials (83 percent) were terminated for futility or lack of efficacy. None was terminated for safety concerns alone, though elenbecestat documented an adverse event profile worse than placebo in addition to lack of efficacy. Two trials were terminated by sponsor decision following pre-planned futility analyses. At the time of the BACE inhibitor clinical trial launches, peer-reviewed literature had already raised mechanistic concerns about BACE1 inhibition that were not incorporated into trial designs.

**Table 2:** 

**Table 2.** Largest terminated Phase 2/3 Alzheimer's disease trials (N ≥ 1,300) with termination reasons and alternative frameworks available at trial initiation

NCT Number	Drug	Mechanism	Phase	N	Termination Reason	Alternative Framework available at initiation
NCT01931566	Pioglitazone	Metabolic (PPAR-γ)	3	3,494	Lack of efficacy; no safety concern	Heneka MT, Lancet Neurology. 2015
NCT02245737	Lanabecestat	Amyloid (BACE)	2/3	2,218	Futility—unlikely to meet primary endpoint	Vassar R, Alzheimers Res Ther. 2014
NCT02956486	Elenbecestat	Amyloid (BACE)	3	2,212	Unfavorable risk-benefit; AEs worse than placebo	Vassar R, Alzheimers Res Ther. 2014
NCT01739348	Verubecestat	Amyloid (BACE)	2/3	2,211	No cognitive benefit; increased AEs	Soscia SJ, PLoS ONE. 2010
NCT01900665	Solanezumab	Amyloid (mAb)	3	2,129	Did not meet primary endpoint	Soscia SJ, PLoS ONE. 2010
NCT02783573	Lanabecestat	Amyloid (BACE)	3	1,722	Futility—unlikely to meet primary endpoint	Vassar R, Alzheimers Res Ther. 2014
NCT04241068	Aducanumab (EMBARK)	Amyloid (mAb)	3	1,696	Sponsor's decision	Guerreiro R, NEJM. 2013
NCT02484547	Aducanumab (EMERGE)	Amyloid (mAb)	3	1,643	Futility—not based on safety concerns	Singhrao SK, J Alzheimers Dis. 2014
NCT02477800	Aducanumab (ENGAGE)	Amyloid (mAb)	3	1,653	Futility—not based on safety concerns	Soscia SJ, PLoS ONE. 2010
NCT01127633	Solanezumab (ext.)	Amyloid (mAb)	3	1,457	Did not meet primary endpoint	Soscia SJ, PLoS ONE. 2010
NCT01953601	Verubecestat (prodromal)	Amyloid (BACE)	3	1,454	No benefit in prodromal AD (Feb. 2018)	Vassar R, Alzheimers Res Ther. 2014
NCT04374253	Gantenerumab (OLE)	Amyloid (mAb)	3	1,382	Terminated following GRADUATE I and II results	Heneka MT, Lancet Neurology. 2015

**Source:** ClinicalTrials.gov. Alternative framework citations from peer-reviewed literature with publication dates predating each trial's start date. AEs: adverse events.

### Temporal alignment: Published alternatives were available

**Figure 3:**
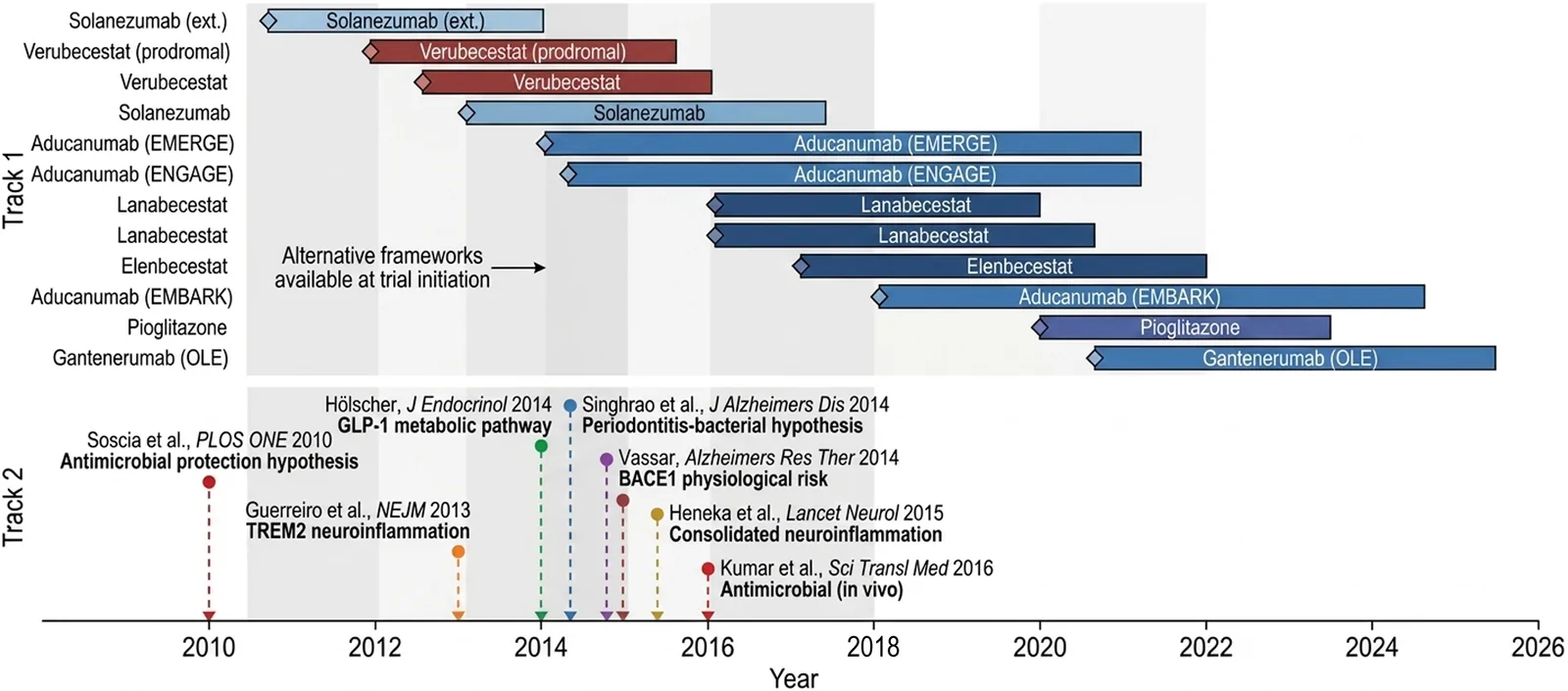
**Figure 3**. **Temporal alignment of the 12 largest Alzheimer's disease clinical trials and key alternative pathogenic framework publications.** Horizontal timeline from 2009 to 2026 showing the active periods of the 12 largest Phase 2/3 Alzheimer's trials by enrollment (N ≥ 1,300) aligned with publication dates of peer-reviewed alternative mechanistic frameworks. Each bar represents a trial's duration; publication markers (dashed vertical lines) indicate when alternative frameworks appeared in the literature. All 12 trials were initiated after at least one alternative framework had been published, yet none of these frameworks was incorporated into trial design, endpoint selection, or Go/No Go deliberations. Alternative frameworks shown include the antimicrobial protection hypothesis (Soscia et al., 2010; Kumar et al., 2016), TREM2 neuroinflammation pathway (Guerreiro et al., 2013), GLP-1 metabolic pathway (Hölscher, 2014), periodontitis-associated bacterial pathogenesis (Singhrao et al., 2014), BACE1 physiological risk prediction (Vassar, 2014), and consolidated neuroinflammation hypothesis (Heneka et al., 2015). Source: ClinicalTrials.gov; peer-reviewed literature.

All 12 trials were initiated after at least one peer-reviewed alternative mechanistic framework had been published (**Figure 3**). Critically, while the sponsors' Investigational New Drug (IND) updates would have mandated literature reviews capturing these competing hypotheses, the regulatory framework (21 CFR 312.23, 312.33) did not require them to justify their target selection over those alternatives.

The solanezumab extension trial (NCT01127633) began in December 2010, nine months after Soscia and colleagues demonstrated in PLOS ONE that amyloid-beta possesses antimicrobial properties in vitro (Soscia et al., 2010)—suggesting that amyloid might be a protective response rather than a primary pathological agent. The verubecestat trial (NCT01739348) began in November 2012, one year before Guerreiro and colleagues identified TREM2 variants as Alzheimer's risk factors in the New England Journal of Medicine (Guerreiro et al., 2013). The lanabecestat trial (NCT02245737) began in September 2014, the same year Vassar published mechanistic concerns about BACE1's physiological necessity for axon guidance and synaptic health (Vassar, 2014)—a prediction that elenbecestat's adverse event profile would later confirm. The aducanumab EMERGE trial (NCT02484547) began in September 2015, two years after the TREM2 discovery and one year after Singhrao established the periodontitis-Alzheimer's association (Singhrao et al., 2014). The 2016 BACE trials began after Heneka's comprehensive neuroinflammation review in *Lancet Neurology* had already laid out the case for inflammation as a primary rather than secondary driver (Heneka et al., 2015).

In every case, the alternative framework predated the trial initiation. In no case did the trial design incorporate the alternative framework's biomarkers, mechanistic assumptions, or endpoint criteria. The Go/No Go meetings that launched these trials were not held in an evidentiary vacuum. They were held in the presence of published, peer-reviewed evidence pointing in a different direction—evidence that was not consulted, not incorporated, and apparently not considered disqualifying.

### The 2015–2016 peak: Maximum investment, maximum available evidence

The heaviest enrollment concentration in the failed trials occurred between 2015 and 2016. This is precisely the period during which the neuro-inflammation case was most conspicuous in the literature. Heneka's consolidated neuro-inflammation review appeared in *Lancet Neurology *in 2015 (Heneka et al., 2015). Kumar and colleagues' *in vivo *confirmation of the antimicrobial protection hypothesis appeared in *Science Translational Medicine* in 2016 (Kumar et al., 2016). Despite these high-impact publications, sponsors launched the lanabecestat trial (NCT02783573, July 2016) and the elenbecestat trial (NCT02956486, October 2016)—the third and fourth BACE inhibitors to enter large-scale Phase 3 testing. Both terminated for futility.

The persistence of the amyloid framework into 2020 is more striking still. The aducanumab EMBARK extension (NCT04241068) began enrollment in March 2020, after the March 2019 public futility signal from the EMERGE and ENGAGE trials had already been announced. The gantenerumab long-term open-label extension (NCT04374253) began in 2020 and terminated following the negative GRADUATE I and II results. A publicly announced futility signal did not halt these extensions. Establishment inertia, not scientific evidence, appears to have been driving the decisions.

### Post-2022: The alternatives also did not produce clinical benefit

The 2022–2026 period extended the negative record into mechanisms that had been proposed as genuine alternatives to amyloid. The simufilam trial (Cassava Sciences, REFOCUS-ALZ, N = 1,125) tested filamin A binding as a non-amyloid, non-tau target and failed unambiguously on both co-primary endpoints. The semaglutide Phase 3 program (Novo Nordisk, EVOKE and EVOKE+, N = 3,808) tested GLP-1 receptor agonism on the strength of compelling observational evidence that diabetes patients on GLP-1 agonists had reduced dementia risk; the trials produced biomarker improvements but no cognitive benefit. This result dealt a substantial blow to the 'Type 3 diabetes' hypothesis—first formally proposed in 2005—which had framed Alzheimer's as a brain-specific insulin-resistant state. AL002 (Alector/AbbVie, INVOKE-2), a TREM2 agonist antibody, showed no clinical benefit and no effect on amyloid, tau, or neurodegeneration biomarkers despite confirmed target engagement. Posdinemab (Johnson & Johnson), an anti-tau antibody, was terminated in November 2025. Nivisnebart (GSK/Alector), targeting the progranulin/sortilin pathway, was stopped for futility in April 2026.

These failures are not footnotes. They are the dataset's most important findings. The field eventually tested alternatives—at scale, with adequate power, with biomarker evidence of target engagement. The alternatives also failed to demonstrate clinical benefit. This result does not vindicate the amyloid hypothesis. It demands a harder question: is the problem the mechanism, or is the problem something more fundamental about how Alzheimer's disease has been defined, staged, and measured?

## Discussion

### What the Go/No Go record reveals

Each trial in this dataset represents a decision point within a development program. For those programs that advanced from Phase 2 to Phase 3, these were formal Go/No Go decisions based on a review of the available Phase 2 clinical evidence. By 2014, the amyloid hypothesis had accumulated enough negative Phase 3 data to constitute a pattern visible to anyone who chose to look. By 2016, the pattern was unmistakable. Yet the Go decisions continued. The third BACE inhibitor entered Phase 3 after the first two had failed to demonstrate benefit. The fourth entered after the third had failed. Aducanumab's extension trial began enrollment after the drug's own futility signal had been publicly announced. These decisions reflect the organizational pressures and incentive structures that systematically favored continuation over termination, regardless of what the scientific evidence demanded.

The question that should have been central to every one of those meetings is not complicated: does the Phase 2 clinical signal for this mechanism, in this patient population, justify a Phase 3 investment of this scale—and does it remain justifiable given the cumulative record of prior trials in the same mechanism class? If the honest answer was no, the Go decision was indefensible. If the honest answer was that no one in the room wanted to ask the question out loud, that is the institutional failure this article documents.

The BACE inhibitor program is the clearest illustration of this failure. Vassar explicitly raised mechanistic concerns in the peer-reviewed literature that BACE1 performs physiological functions essential for axon guidance and synaptic integrity—functions that BACE inhibition would disrupt regardless of its effect on amyloid (Vassar, 2014). That mechanistic concern was published before the third and fourth BACE inhibitor trials were launched. It was not incorporated into the trial designs, not reflected in the endpoint selection, and apparently not considered disqualifying at the Go decision point. Elenbecestat's adverse event profile—worse than placebo—confirmed the prediction. The sponsors had been informed of the mechanistic concern in print. They proceeded anyway. **Figure 1** illustrates one way of formalizing this vagueness and ambiguity in Go/No Go trial decisions: treating subjective uncertainty, partial truths, lack of knowledge, and conflicting evidence as probabilities assigned to subsets of possibilities rather than to single outcomes.

### Three establishment mechanisms that produced predictable outcomes

The Go/No Go decisions were not random, and they were not primarily scientific. They were the predictable output of three institutional forces operating simultaneously on decision-makers who had every organizational incentive to proceed and very little protection if they chose to stop.

Regulatory path dependency is the most structural explanation. Once the Food and Drug Administration accepted amyloid clearance as a surrogate endpoint for accelerated approval, sponsors had a rational incentive to follow that pathway regardless of its scientific merit. A novel mechanism—TREM2, periodontitis-associated bacteria, GLP-1—would face not only the scientific uncertainty inherent in any Phase 3 trial but also the regulatory uncertainty of establishing an entirely new evidentiary pathway. Amyloid, despite its uniformly poor efficacy record, offered known regulatory ground. Regulatory precedent, not scientific evidence, was driving mechanism selection.

Institutional entrenchment is the second mechanism. NIH grant review panels, journal editorial boards, and industry scientific advisory boards have been disproportionately populated by researchers whose careers and reputations were built within the amyloid framework. This is not a conspiracy but a predictable outcome of scientific path dependency: dominant paradigms tend to reproduce themselves through gatekeeping systems. Researchers exploring alternative frameworks have faced higher evidentiary barriers, regardless of the quality of their data.

Shareholder pressure and sunk-cost entrenchment is the third and most direct force. A pharmaceutical executive who terminates a billion-dollar program on the basis of a Phase 2 signal that the field's review panels have already endorsed faces immediate consequences: investor calls, board scrutiny, and the accusation of having wasted everything invested to that point.

An executive who proceeds to Phase 3 and fails can blame the biology. The asymmetry of personal and organizational consequences systematically favored continuation over termination, regardless of what the scientific evidence demanded. Amyloid, despite its catastrophic failure record, offered the further advantage of a known regulatory pathway and an established surrogate endpoint. A sponsor defending a Go decision to shareholders could point to FDA precedent. A sponsor proposing to pivot to neuroinflammation or periodontitis-associated bacterial pathways could not. The result was a field in which the incentives for continuing a failed approach were stronger than the incentives for abandoning it—and in which the patients enrolled in the resulting trials paid the price.

### The counter-establishment perspective: George Perry and the Journal of Alzheimer's Disease

The concentration of resources around the amyloid framework has been recognized and contested within the field itself. Investigative journalist Charles Piller, in his book *Doctored* (2025a) and his subsequent reporting for STAT News (2025b), documented what he and others have termed the “amyloid mafia”—a network of researchers whose dominance of grant review panels, journal editorial boards, and regulatory advisory committees has, in critics' view, systematically starved alternative hypotheses of funding. Zaven Khachaturian, former director of Alzheimer's research at the National Institute on Aging, has described the field's convergence on amyloid as having hardened into an unquestioned orthodoxy that scientists felt obligated to defer to rather than interrogate (Piller, 2025a, 2025b).

This characterization is contested, and the contest itself is instructive. Dennis Selkoe, one of the most prominent proponents of the amyloid hypothesis, has published a direct rebuttal arguing that researchers converged on amyloid because the evidence for its causal role—including genetic mutations in amyloid precursor protein (APP) and the consistent slowing of decline across anti-amyloid trials—is genuinely strong, not because of gatekeeping (Selkoe, 2025). Selkoe's position is that scientific consensus tracks evidence, not enforcement.

The career and editorial strategy of George Perry, Editor-in-Chief of the Journal of Alzheimer's Disease (JAD), offers a case study in how counter-establishment perspectives have fared. Perry has been one of the earliest and most persistent critics of the dominance of the amyloid hypothesis, arguing that amyloid-beta accumulation is not a primary toxic driver but a downstream, protective antioxidant response (Zhang, 2017). In Perry's model, the brain deploys amyloid-beta as a shield to encapsulate threats and manage oxidative stress; removing this shield through anti-amyloid antibodies may dismantle the brain's defensive structures while leaving the initial trigger intact. This prediction is consistent with the absence of correlation between amyloid removal and clinical benefit observed in lecanemab and donanemab trials.

Perry's decision to establish the Journal of Alzheimer's Disease as a platform for alternative perspectives—including the infectious hypothesis, metabolic models, and the protective amyloid theory—was itself a response to what he and his colleagues described as systematic difficulty publishing counter-establishment work in mainstream journals. Perry has noted that his trainees and colleagues faced negative feedback from grant reviewers for their association with him (Zhang, 2017). Whether this reflects gatekeeping or merely the normal operation of peer review is disputed, but the fact of the perception—and the existence of JAD as an alternative forum—is itself documented evidence of institutional friction within the field.

The 567-trial record assembled here offers a way to adjudicate the dispute empirically. If Selkoe's account is correct—that convergence reflects the weight of evidence—the trial record should show a field self-correcting as disconfirming data accumulated. If the critics' account is correct— that convergence reflects institutional path dependency—the record should show repeated Go decisions on a mechanism class despite mounting negative Phase 3 evidence. The data presented in this analysis are more consistent with the latter pattern than the former.

### Why did the alternatives also fail?

The post-2022 failures present the hardest intellectual challenge this dataset poses. If the problem were simply amyloid-centric and establishment inertia, one would expect those well-powered trials of genuinely novel mechanisms—TREM2 agonism, GLP-1 receptor stimulation, progranulin restoration—would eventually produce different results. They have not. Three non-exclusive explanations warrant serious consideration.

**Explanation 1: Disease heterogeneity.** Alzheimer's disease, as currently defined by clinical and biomarker criteria, may not be a single disease but a clinical syndrome produced by multiple distinct underlying pathologies that share a phenotypic presentation. A Phase 3 trial designed to test one mechanism in a population defined by amyloid PET positivity and cognitive decline will necessarily include patients for whom that mechanism is irrelevant to their particular disease trajectory. The dilution of effect in a heterogeneous population cannot be compensated for by larger sample sizes. It requires better patient stratification, which in turn requires better understanding of which patients have which underlying pathology.

Evidence from the 567-trial dataset suggests that aggregated enrollment frequently masks pathologically meaningful subgroup differences, leading to 'mechanism failure' labels for what are population-selection errors. This dilution is evidenced by the APOE4-specific biomarker dissociation in bapineuzumab trials, the contradictory outcomes of the identical EMERGE/ENGAGE aducanumab studies, and the directionally opposite effects seen in mild vs. moderate populations in the NILVAD trial (Lawlor et al., 2018). Together, these examples suggest that the null results littering the 567-trial dataset may frequently reflect the dilution of real mechanistic effects in patients who were never meaningfully separable by the enrollment criteria applied. These cases illustrate that when a single composite endpoint is applied to a biologically heterogeneous population, even potent mechanisms will produce null results that cannot be rescued by larger sample sizes, only by precise stratification.

**Explanation 2: Intervention timing.** Virtually all the mechanisms being tested—amyloid clearance, tau reduction, TREM2 activation, GLP-1 receptor stimulation—may be intervening downstream of the true initiating insult, or at a disease stage when the window for modification has already closed. The Brain study finding that one-third of non-demented individuals with full amyloid and tau pathology never develop dementia suggests that the pathology precedes the disease by a substantial margin. Intervening at symptom onset may be as futile as treating a fever after the infection has already caused irreversible organ damage.

**Figure 4:**
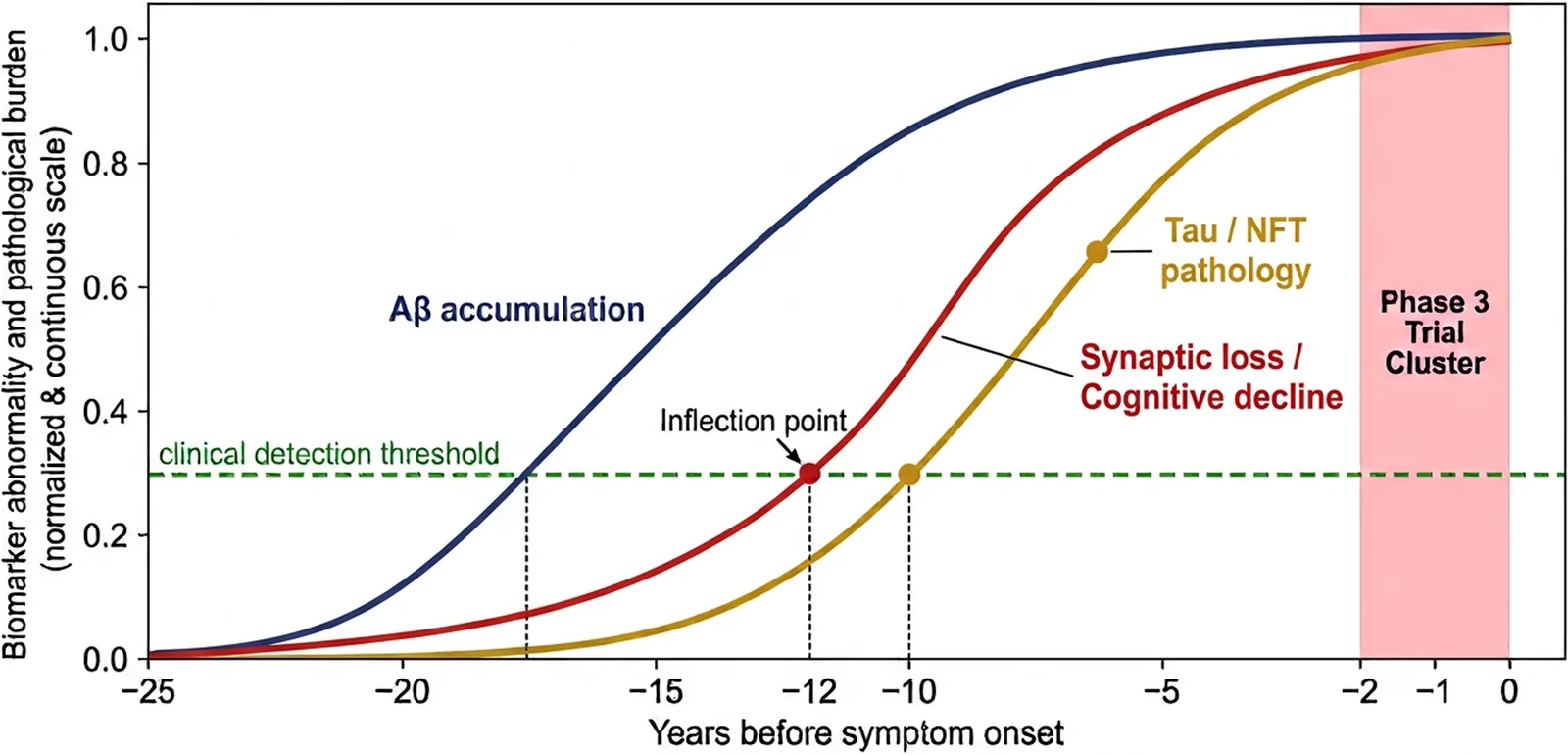
**Figure 4**. **Synthetic biomarker cascade based on Bateman et al. (2012) and Jack et al. (2013)**. Phase 3 trial cluster derived from ClinicalTrials.gov dataset (N = 189 Phase 3 AD trials, start years 2000–2024). Curves are illustrative and not derived from individual participant data. The emphasized red curve (synaptic loss) accelerates a full decade before typical Phase 3 enrollment, indicating that intervention at symptom onset targets a brain that has already passed the inflection point of homeostatic recovery.

The empirical basis for this “too-late” explanation is the DIAN longitudinal biomarker dataset. Bateman et al. (2012) demonstrated in mutation carriers of dominantly inherited Alzheimer's disease that cerebrospinal fluid Aβ42 begins to decline approximately 25 years before expected symptom onset, with amyloid PET abnormalities detectable 15 years before onset, tau accumulation at 10–15 years, hippocampal atrophy at five years, and clinically measurable cognitive impairment appearing last (Bateman et al., 2012). By the time a patient meets the enrollment criteria typical of trials in this dataset—symptomatic mild cognitive impairment (MCI) or early dementia with amyloid PET confirmation—the brain has endured one to two decades of proteopathic stress. **Figure 4** illustrates the temporal misalignment between the 25-year biomarker cascade and the 18-to-24-month window of the Phase 3 trial cluster, highlighting how legacy interventions have historically targeted the final symptomatic stage of a multi-decade pathological process.

The 25-year biomarker cascade modeled in **Figure 4** utilizes sigmoidal functions to reflect the non-linear kinetics of proteopathic accumulation. In this framework, long periods of sub-clinical stability are followed by a phase of exponential pathological acceleration—the kinetic inflection point—before reaching a plateau of terminal neurodegeneration. Crucially, the synaptic-loss/cognitive-decline curve in **Figure 4** identifies the inflection point for synaptic loss and neurodegeneration, which accelerates a full decade prior to the typical symptomatic enrollment window. A disease-modifying drug would have its highest probability of demonstrating clinical efficacy if administered to each patient immediately prior to this sharp acceleration on the sigmoid curve, while the biological system still possesses the capacity for homeostatic recovery and synaptic repair.

For the neuropathologist, this non-linear progression is an indictment of trial timing: it demonstrates that by the time the 'Phase 3 Cluster' (2000–2024) initiates intervention, the brain has already transitioned from a manageable seeding phase to a saturated state of irreversible synaptic rarefaction. By intervening at the plateau of the synaptic-loss/cognitive-decline curve rather than its inflection point, legacy trials have effectively attempted to arrest a process that has already reached its pathological zenith. This mismatch shifts the narrative from failures of psychological scales like the Alzheimer's Disease Assessment Scale–Cognitive Subscale to a predictable biological reality—the attempt to repair a circuit that has already been fundamentally dismantled.

The A4 study (Anti-Amyloid Treatment in Asymptomatic Alzheimer’s Disease, NCT02008357) tested this logic prospectively, enrolling cognitively normal amyloid-positive adults and randomizing them to solanezumab over 4.5 years; the result was negative, but solanezumab targets soluble monomeric amyloid without clearing fibrillar plaques—a mechanism limitation independent of the timing question. A4's failure is at minimum ambiguous between “too early” and “wrong target form.”

The field's prevention trials constitute an implicit acknowledgment of the timing problem, and the DIAN-TU gantenerumab extension provides the most direct evidence bearing on it. The original randomized trial had failed to slow cognitive decline in a mixed symptomatic and asymptomatic population; the open-label extension, reported in The Lancet Neurology in March 2025, followed 22 asymptomatic mutation carriers receiving high-dose gantenerumab for an average of eight years and found an approximately 50 percent reduction in dementia progression risk relative to external controls, with an estimated three to six years' delay in symptom onset (Bateman et al., 2025).

The finding is not confirmatory. The extension was open-label, the asymptomatic subgroup was small, and controls were external rather than randomized. It is, however, the first clinical trial result consistent with the hypothesis that anti-amyloid treatment before symptom onset can delay dementia, using the same antibody that had already failed when given after symptoms appeared. AHEAD 3-45 (NCT04468659) takes this logic forward, enrolling cognitively unimpaired adults stratified by amyloid burden—a design that instantiates both the timing correction and the stratification-first approach that Explanation 1 demands.

**Explanation 3: Endpoint mismatch.** The field's endpoint selection may be systematically misaligned with what the proposed mechanisms actually do on the timescales of 18-month Phase 3 trials. The semaglutide trials are the clearest example: the drug produced measurable improvements in neuroinflammation biomarkers but no detectable cognitive benefit within the trial window. This is not necessarily evidence that GLP-1 receptor agonism is ineffective against Alzheimer's pathology—it may be evidence that the biomarkers being measured do not predict the clinical outcomes being sought, or that the mechanisms being targeted operate on timescales longer than standard Phase 3 trials can capture.

One mechanistic explanation for why amyloid clearance specifically may cause harm rather than benefit has been outlined in a companion publication. If amyloid plaques and tau tangles are part of evolutionarily conserved innate immune responses—the brain's way of isolating pathogens or inorganic nucleation seeds—then anti-amyloid antibodies do not actually treat the disease. Instead, they dismantle the brain's defensive structures while leaving the initial trigger intact. Under this framework, Amyloid-Related Imaging Abnormalities seen in lecanemab and donanemab trials likely represent an inflammatory rebound from that dismantling, rather than drug toxicity. This prediction explains why the extent of amyloid removal does not correlate with clinical improvement. The complete mechanistic argument is detailed elsewhere (Guth, 2026).

None of these explanations is comfortable. All of them suggest that the problem is deeper than mechanism selection, and that the field requires not only different targets but different trial designs, different patient populations, earlier intervention windows, and endpoint definitions that are sensitive to the proposed mechanism's effects.

### The field's unfinished reckoning

Pharmaceutical firms have sponsored so many clinical trials of anti-amyloid agents, with little or no hint of efficacy, that they have long passed the definition of insanity: doing the same thing over and over in the hope of getting a different result. That observation applied to the state of the art even a decade or two before the aducanumab EMBARK extension began enrollment.

The field has not yet completed the reckoning that the 567-trial record demands. It is not enough to acknowledge that amyloid trials failed to demonstrate clinical benefit. The harder acknowledgment—one the post-2022 data now makes unavoidable—is that the alternatives also failed. The field spent thirty years insisting the problem was amyloid-centric. The post-2022 data shows that removing that focus was not sufficient. Mechanisms with genuinely different targets, tested at adequate scale with biomarker confirmation of target engagement, also failed to demonstrate clinical benefit. This means the problem is not only which mechanism is chosen. It is whether the field is testing drugs early enough, in the right patients, and measuring outcomes that are actually sensitive to what disease modification would look like on a biologically realistic timescale.

As quoted in Sullivan (2019), Michael S. Wolfe, Ph.D., observed: “To me, the failures of the anti-amyloid approaches are because the drugs are given too late, are targeting the wrong form of Aβ, or are targeting an enzyme [for example, beta secretase 1] that has other important functions. Most likely it's a combination of these reasons. One could argue that even if some form of Aβ is the pathogenic entity, it is not a practical target because intervention may need to be initiated many years before the onset of symptoms.”

That reckoning has now begun within the field itself. Li et al.—with Sperling, Aisen, and Donohue among the co-authors—analyzed divergent cognitive trajectories among amyloid-positive participants in preclinical trials. They concluded that enrolling large numbers of stable amyloid-positive individuals dilutes overall treatment effects and reduces a trial's ability to detect meaningful cognitive benefit. Statistical power is driven primarily by the declining minority, not the enrolled majority (Li et al., 2026). The authors were careful to note that the goal was not homogeneous enrollment but transparency about the inefficiency introduced when multiple latent trajectory classes occupy a single trial cohort—the precise inefficiency that the 567-trial record instantiates at scale.

**Figure 5:**
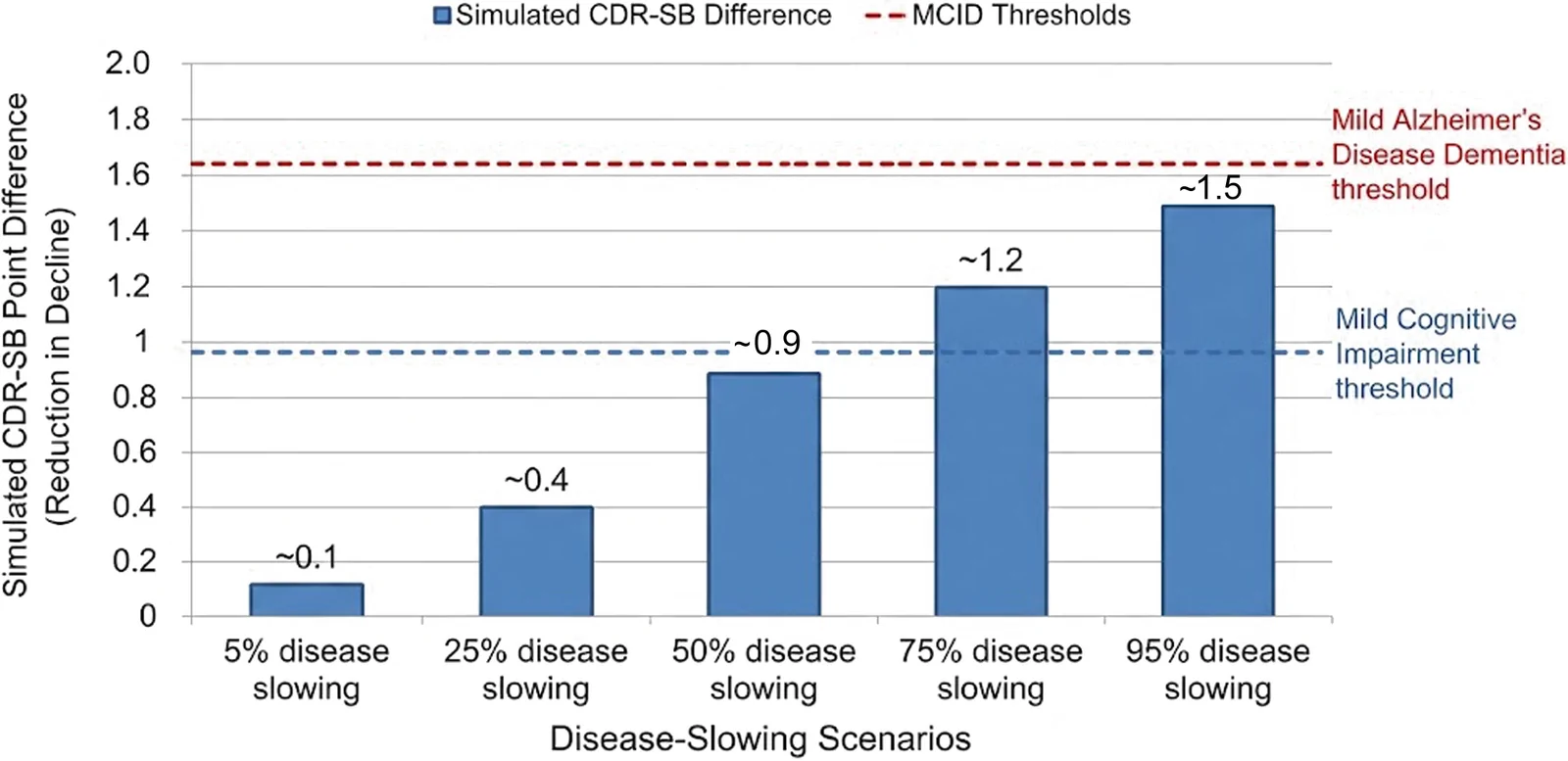
**Figure 5**. **Endpoint mismatch**: Published MCID thresholds vs. expected effects of disease modification. Bar chart comparing simulated CDR-SB (Clinical Dementia Rating Scale—Sum of Boxes) point differences across disease slowing scenarios (5 percent to 95 percent) from Lanctôt et al. (2025) against published minimal clinically important difference (MCID) thresholds for mild Alzheimer's disease dementia (1.0–1.5 points). Even complete halting of disease progression may not consistently meet MCID thresholds in all populations or trial durations. This structural mismatch suggests that a substantial portion of the null results in the 567-trial dataset may reflect not the absence of biological effect but the incapacity of the endpoint standard to register clinically meaningful change within typical 18-month trial windows. Simulations from Lanctôt et al. (2025). MCID thresholds from published literature.

As with the timing problem, the endpoint problem has now been quantified directly. Lanctôt et al. simulated scenarios of 5 to 95 percent disease slowing using Alzheimer's Disease Neuroimaging Initiative (ADNI) data and compared resulting Clinical Dementia Rating – Sum of Boxes (CDR-SB) point differences against published minimal clinically important difference (MCID) thresholds. For mild AD dementia, realistic treatment effects (e.g., 25 % to 50 % slowing) fall well below or only marginally graze published MCID thresholds, meaning that meaningful biological modification yields point differences often categorized as 'not clinically meaningful.' Even near-complete halting of progression yields differences that struggle against conservative threshold bounds, leading the authors to conclude that published MCIDs in AD are frequently incompatible with the expected incremental benefits of early disease modification. Applying them to between-group trial results erroneously classifies months of preserved cognitive function as non-meaningful (Lanctôt et al., 2025). The implication for the 567-trial record is precise: a substantial portion of the null results in that dataset may reflect not the absence of biological effect but the structural incapacity of the endpoint standard to register one. **Figure 5** illustrates the endpoints related to MCID.

These three explanations are not mutually exclusive, and the evidence suggests all three are operating simultaneously.

The central lesson of the 567-trial dataset is not subtle. Do not repeat a mechanism of action that the published peer-reviewed literature has already called into question. Do not repeat a mechanism class that prior Phase 3 trials in the same dataset have already falsified. Do not allow the absence of a better candidate, the size of the sunk investment, or the fear of shareholder reaction to override what the evidence is telling you. Six million Americans and 35 million people worldwide are waiting for a drug that addresses the disease rather than its downstream markers. They cannot afford another generation of Go decisions driven by institutional momentum rather than scientific integrity.

## Conclusion

The 567-trial dataset of Phase 2 and Phase 3 Alzheimer's trials from 2010 to 2026 is the most comprehensive empirical record of concentrated therapeutic failure in modern medicine. The field-tested amyloid clearance at scale and failed. It tested non-amyloid mechanisms at scale and failed. The common factors across both sets of failures are not limited to mechanism selection. This record points to three deeper, interrelated problems: disease heterogeneity, intervention timing, and endpoint mismatch.

The DIAN longitudinal biomarker data establishes that Alzheimer's pathology begins 15–25 years before symptom onset (Bateman et al., 2012). Every Phase 3 trial in this dataset intervened after symptoms appeared. The 2025 DIAN-TU open-label extension provides the first evidence that anti-amyloid treatment before symptom onset may delay dementia—using the same antibody that failed when given after symptoms appeared (Bateman et al., 2025).

The practical barriers to prevention are substantial: biomarker stratification is imperfect, long-term treatment is expensive, and most at-risk individuals will never develop dementia. Moreover, the upstream drivers of neurodegeneration may include social stressors—unemployment, housing insecurity, chronic trauma—that no monoclonal antibody can address. These are not scientific objections; they are operational and societal challenges. But they are not reasons to continue intervening after symptom onset, which has been tested on 567 occasions. They are reasons to confront the deeper question of what Alzheimer's prevention actually requires, and whether the current research enterprise is capable of delivering it.

What is required is not another advisory panel—such bodies have proven susceptible to the same institutional forces that produced the 567-trial record. What is required is a structural reallocation: at least 50 percent of Alzheimer's research funding should be directed to prevention trials in asymptomatic at-risk populations, with long-term follow-up (> 10 years). This approach recognizes that the alternative—intervening after symptom onset—has been tested at scale and found uniformly ineffective. The specific prevention strategy remains to be determined. What is not debatable is that continuing the 18-month Phase 3 model for symptomatic disease, after the 567-trial record, cannot be justified by any reading of the evidence.

The patients enrolled in the next generation of trials, and the millions worldwide who will never enroll in any trial, cannot afford another three decades of the same answer.

## Funding statement

The author received no financial support for the research, authorship, and/or publication of this article.

## Data availability

No original experimental data were generated during the preparation of this article. The analytic dataset was compiled from publicly available ClinicalTrials.gov records and published literature. The trial-level dataset/classification filters are found at ClinicalTrials.gov.

## Author contributions

MASG: Conceptualization, Investigation, Writing — Original Draft, Writing — Review & Editing. The author researched the clinical trial record for drugs tested to treat Alzheimer's disease and found supporting and contradicting peer-reviewed literature.

## AI contribution

During the preparation of this manuscript, the author used large language models (DeepSeek, Gemini, and Claude) for language refinement, grammar checking, and assistance with literature synthesis in response to reviewer comments. Prior to submission, DeepSeek, Gemini and Claude functioned as critic-reviewers pointing out errors or revisions needed in wording through three prior drafts. The author reviewed and edited all AI-generated suggestions and assumes full responsibility for the final content. Gemini 3 created each of the five figures in this article, which were described and refined by the author.

## Conflict of interest statement

The author, Michael A. S. Guth, declares no financial or commercial conflicts of interest. In alignment with the ethos of Free Neuropathology, the author affirms that this work was conducted in total intellectual independence. There has been no influence, funding, or oversight from pharmaceutical entities involved in clinical trials and drug development related to Alzheimer's disease. This manuscript serves solely as an independent synthesis of the published evidence, unencumbered by the institutional or corporate biases that frequently govern neurodegenerative research.
